# Sedentary behavior, physical activity, sleep duration and obesity risk: Mendelian randomization study

**DOI:** 10.1371/journal.pone.0300074

**Published:** 2024-03-08

**Authors:** Siqing Chen, Lili Yang, Yuting Yang, Wenmini Shi, Matthew Stults-Kolehmainen, Qiao Yuan, Chenchen Wang, Jing Ye

**Affiliations:** 1 Department of Nursing, International Institutes of Medicine, The Fourth Affiliated Hospital, Zhejiang University School of Medicine, Yiwu, Zhejiang, China; 2 Department of Biobehavioral Sciences, Teachers College-Columbia University, New York, NY, United States of America; 3 Li Ka Shing Faculty of Medicine, School of Public Health, The University of Hong Kong, Hong Kong, SAR China; 4 Center for Weight Management, Digestive Health Multispecialty Clinic, Yale New Haven Hospital, New Haven, CT, United States of America; University of Costa Rica, COSTA RICA

## Abstract

**Background:**

Observational studies have suggested associations between sedentary behaviors (SB), physical activity (PA), sleep duration (SD), and obesity, but the causal relationships remain unclear.

**Methods:**

We used Mendelian randomization (MR) with genetic variation as instrumental variables (IVs) to assess the causality between SB/PA/SD and obesity. Genetic variants associated with SB/PA/SD were obtained from Genome-wide association study (GWAS), and obesity data came from FinnGen. The primary MR analysis used the instrumental variable weighted (IVW) method, with sensitivity tests including Cochran Q, MR-Egger intercepts, and MR-Radial. Expression Quantitative Trait Loci (eQTL) analysis was applied to identify significant genetic associations and biological pathways in obesity-related tissues.

**Results:**

The MR analysis revealed causal relationships between four SB-related lifestyle patterns and obesity. Specifically, increased genetic liability to television watching (IVW MR Odds ratio [OR] = 1.55, [95% CI]:[1.27, 1.90], *p* = 1.67×10^−5^), computer use ([OR] = 1.52, [95% CI]:[1.08, 2.13], *p* = 1.61×10^−2^), leisure screen time (LST) ([OR] = 1.62, [95% CI] = [1.43, 1.84], *p* = 6.49×10^−14^, and driving (MR [OR] = 2.79, [95% CI]:[1.25, 6.21], *p* = 1.23×10^−2^) was found to increase the risk of obesity. Our findings indicate that no causal relationships were observed between SB at work, sedentary commuting, PA, SD, and obesity. The eQTL analysis revealed strong associations between specific genes (RPS26, TTC12, CCDC92, NICN1) and SNPs (rs10876864, rs2734849, rs4765541, rs7615206) in both subcutaneous and visceral adipose tissues, which are associated with these SBs. Enrichment analysis further revealed that these genes are involved in crucial biological pathways, including cortisol synthesis, thyroid hormone synthesis, and insulin secretion.

**Conclusions:**

Our findings support a causal relationship between four specific SBs (LST, television watching, computer use, driving) and obesity. These results provide valuable insights into potential interventions to address obesity effectively, supported by genetic associations in the eQTL and enrichment analysis. Further research and public health initiatives focusing on reducing specific SBs may be warranted.

## Introduction

World Health Organization (WHO) declared obesity a global epidemic [1]. That affects people of all ages and socioeconomic groups, independent of a country’s income level [2,3]. Over the past few decades, the prevalence of obesity has steadily risen, becoming a significant public health challenge globally [4]. Currently, approximately 30% of adults worldwide are affected by obesity, and this number is projected to increase to 33% by 2030 [5,6]. Obesity is a complex condition with multiple contributing factors, including genetics, behavior, and the environment [7]. It is associated with a higher risk of cancer [8], cardiovascular diseases, and increased mortality rates [9], while also bringing negative psychosocial consequences, such as social stigma and depression. In addition to adverse health effects, obesity takes a considerable toll on the economy, encompassing expenses for preventive measures, diagnostic procedures, pharmaceuticals, and treatment services [10]. Alarmingly, the projected medical costs associated with obesity are estimated to surge annually by a staggering £1.9–2 billion in the UK and a staggering $48–66 billion in the USA by 2030 [11].

Sedentary behavior (SB), characterized by low energy expenditure activities (≤1.5 metabolic equivalents) [12], is a risk factor for obesity [13], and a health burden that influences mortality [14]. Conversely, physical activity (PA) involves musculoskeletal movements requiring additional energy expenditure and has demonstrated numerous health benefits, including improved weight management and a reduced risk of obesity [15,16]. Similarly, sleep duration (SD), an essential component of overall well-being, has been associated with metabolic processes, appetite control, and body weight regulation [17,18]. Rosenberger describes how these are related to the 24-hour activity cycle [19].

Observational studies [15,16,20] indicate that SB, PA, and SD are associated with obesity. For example, high levels of total SB, such as excessive television watching, are associated with obesity across all age groups. In participants who spend three hours or more daily in SB [21], the risk of being overweight or obese increases by 38%. Similarly, some observational studies suggest a connection between higher levels of PA and reduced obesity risk [15,22,23]. Individuals with a sedentary lifestyle have a significantly higher risk of developing obesity [22]. In contrast, young adult females with high levels of PA have a 142% lower likelihood of developing obesity than those with low PA levels [15].

Moreover, some studies [1,24,25] have demonstrated an association between SD and obesity. However, it should be noted that some SB/PA/SD studies have reported conflicting and/or inconsistent findings with the results mentioned above [26–28]. Although observational studies provide valuable insights, they are limited by potential confounding factors and reverse causation, leading to gaps in understanding the causal relationship between SB/PA/SD and obesity. Experimental data on the topic is limited, unfortunately [23].

Mendelian randomization (MR) is a robust approach for establishing causality between exposures and outcomes [29]. It utilizes genetic variants as instrumental variables to minimize confounding [30], ensuring that genetic variations impact obesity development without reverse causality [31]. MR provides higher-quality evidence compared to observational studies, enabling causal inference on the effects of SB/PA/SD on obesity risk [32]. Our study objective was to examine casual associations between lifestyle behaviors (SB, PA, and SD) and obesity outcomes by analyzing large-scale genetic data and using robust analytical methods.

## Methods

### Study design

Our study complied with the standards of the Strengthening the Reporting of Observational Studies in Epidemiology using the Mendelian Randomization S1 Checklist [33].

### Data source

To examine the causal effect of SB/PA/SD on obesity, we first selected ten lifestyle factors strongly associated with obesity based on observational studies. These factors derived from Genome-Wide Association Studies (GWAS) included various forms of SB: television watching (N = 437,887), computer use (N = 360,895), and driving (N = 310,555) [34]. We also incorporated additional GWAS data for leisure screen time (LST) (N = 526,725), covering activities like watching television, playing video games, or sitting in front of a computer; SB at work (N = 372,609), characterized by predominantly sitting and minimal heavy lifting; and sedentary commuting (N = 159,606), which refers to driving a car. Detailed definitions of the three phenotypes can be found in the supplementary data in Zhe Wang study [35]. Furthermore, our study assessed PA factors, including moderate-to-vigorous physical activity (MVPA), accelerometer-based activity with average acceleration (AccAve) (N = 91,084), and physical activity with accelerations over 425 milli-gravities (Acc425) (N = 90,667) [36], as well as sleep duration (SD) (N = 91,105) [36,37]. The data can be found on the GWAS catalog (https://www.ebi.ac.uk/gwas/). Table 1 highlights the Characteristics of data sources.

**Table 1:** Characteristics of data sources.

Phenotype		Consortium	PMID	Author	Decent	Sample size
*Exposure*						
SB	LST	UK Biobank	36,071,172	Wang et al	Eur	526,725
	SB at work				Eur	372,605
	Sedentary commuting				Eur	159,606
	Television watching	UK Biobank	32,317,632	van et al	Eur	437,887
	Computer use				Eur	310,555
	Driving				Eur	422,218
PA	AccAve	UK Biobank	29,899,525	Klimentidis et al	Eur	91,084
	MVPA				Eur	377,000
	Acc425				Eur	91,084
SD		UK Biobank	30,531,941	Doherty et al	Eur	91,105
*Outcome*						
Obesity		FinnGen	36,653,562	Kurki MI et al.	Eur	377,161

Abbreviations: SB, sedentary behavior; PA, physical activity; LST, Leisure screen time; SB at work, Sedentary Behavior at work; AccAve, accelerometer-based physical activity with average acceleration; Acc425, accelerometer-assessed fraction of accelerations>425 milligravities; MVPA, moderate to vigorous physical activity; Eur, European; SD, Sleep duration.

**Fig 1:**
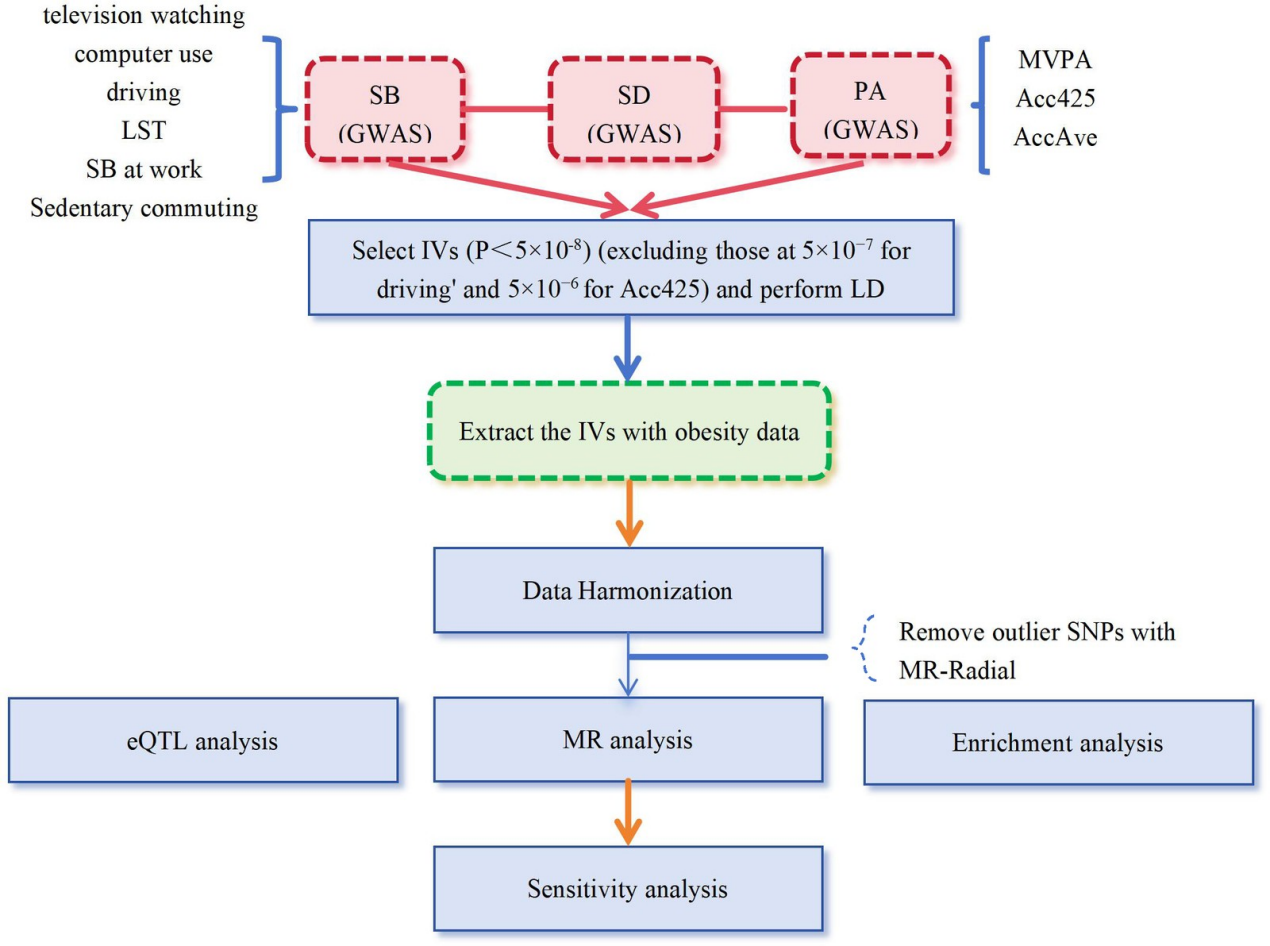
The process fo finstrumental variable selection and study design. SB, sedentary behavior, PA, physical activity; SD, sleep duration; LST, Leisure screen time; SB at work, Sedentary Behavior at work; MVPA, moderate to vigorous physical activity; Acc425, accelerometer-based physical activity with average acceleration; SD, Sleep duration; GWAS: Genome wide association study; IVs: Instrument variables; MR, Mendelian randomization; SNPs: single-nucleotide polymorphisms.

**Fig 2:**
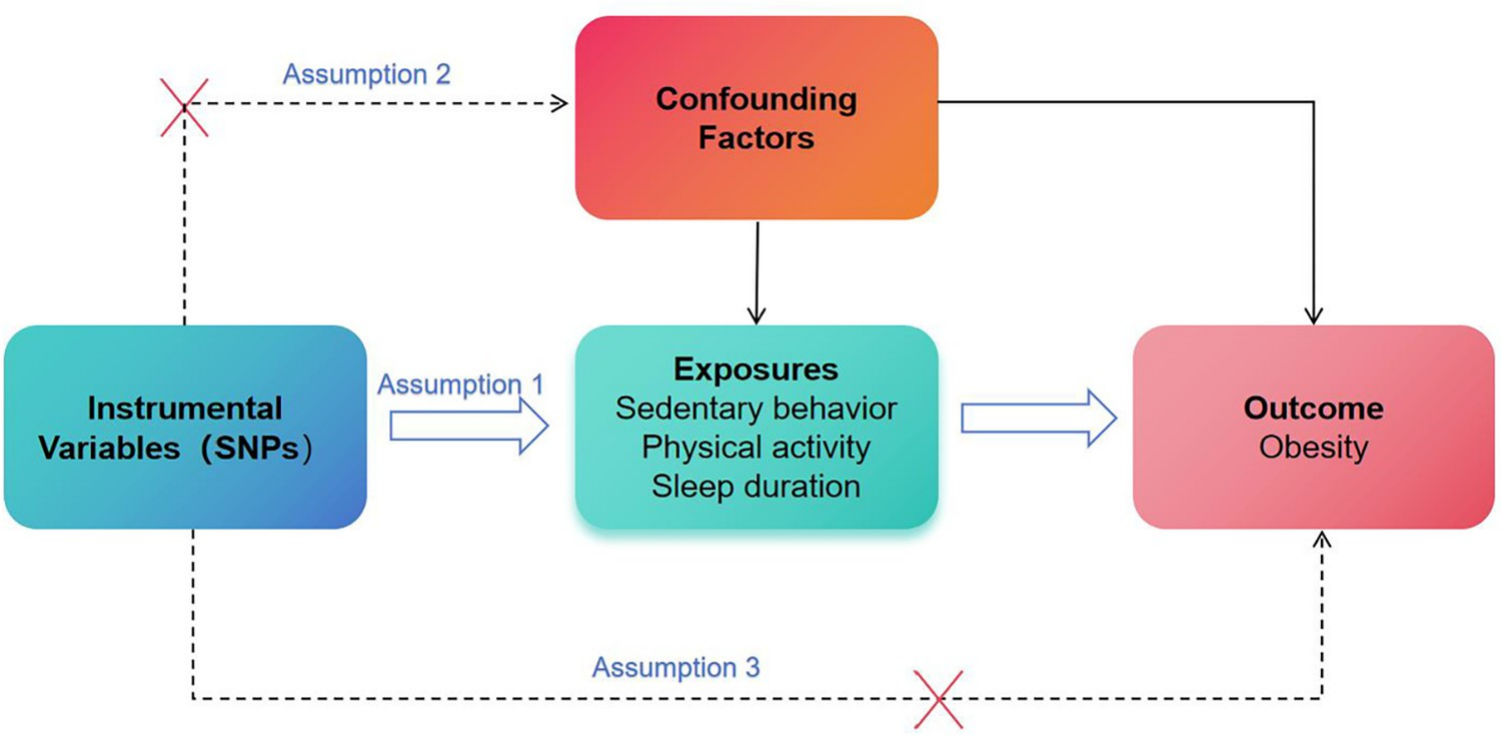
Instrumental variables fit the assumptions of MR. SNPs: single-nucleotide polymorphisms.

We then utilized exposure-related single-nucleotide polymorphisms (SNPs) as instrumental variables (IVs) in our MR study (Fig 1). To ensure the validity of our MR analysis, we selected SNPs to meet three crucial assumptions: 1) strong association between the exposure and SNPs; 2) no association between the SNPs and potential confounding factors; and 3) the SNPs should solely impact the outcome through exposure (Fig 2) [29]. We performed rigorous screening and clumping of the significant SNPs (*p*<5×10^−8^), after we clumped these SNPs in linkage disequilibrium (LD, clumping window: 10000kb, Clumping r^2^ cutoff: 0.001). For phenotypes with fewer than three SNPs, we performed a secondary screening with relaxed *P* value thresholds of less than 5×10^−7^ (driving) and 5×10^−6^ (Acc425) while keeping all other screening conditions unchanged [38]. Ultimately, we identified a set of final SNPs associated with various SBs such as television watching (161 SNPs), computer use (47 SNPs), driving (5 SNPs), LST (115 SNPs), SB at work (9 SNPs), sedentary commuting (16 SNPs), MVPA (19 SNPs), AccAve (8 SNPs), Acc425 (26 SNPs), and SD (14 SNPs). All identified SNPs showed F-statistics greater than 10. Further details about these SNPs are provided in the S2 Table in S2 File.

To avoid any sample overlap bias, we utilized the obesity datasets from FinnGen (https://www.finngen.fi/en), which were updated in December 2022. The FinnGen datasets provided a robust and independent sample population with 21,375 cases and 355,786 controls, all of whom had European ancestry (Table 1).

### MR and sensitivity analysis

To examine the causal effects of PA, SB, and SD on obesity, we employed several statistical methods in our MR study. The Instrumental Variable Weighted (IVW) method and weighted median with MR-Egger, Simple mode, and weighted mode were used for analysis. Depending on the presence of heterogeneity and pleiotropy in IVs, we selected the appropriate analysis approach. When there was no heterogeneity and pleiotropy in IVs, the fixed-effects model analysis using IVW was considered the most accurate. However, the random-effects model of IVW or methods like weighted median was preferred in heterogeneity. Additionally, when SNPs exhibited pleiotropy, MR-Egger analysis was used as an alternative to IVW and weighted median methods [39].

We performed the Cochran Q test to test for heterogeneity, and the funnel plot was used as a supplement. Detecting horizontal pleiotropy, which occurs when instrumental variables directly affect the outcome independent of the exposure factor, is crucial for maintaining the integrity of MR. To address this, we conducted an MR-Egger intercept analysis. Vertical pleiotropy, where an exposure influences an outcome through a common causal pathway, is acceptable for MR inference, unlike horizontal pleiotropy which poses challenges [40], we employed MR-Radial analysis to assess vertical pleiotropy and identify biased SNPs (*p*<0.05). In case biased SNPs were detected, MR and sensitivity analyses were repeated after excluding these SNPs. Additionally, leave-one-out analysis was utilized to assess the robustness of the analysis results [41].

### The eQTL analysis

The Genotype-Tissue Expression (GTEx) project provides the largest atlas of human gene expression and catalog of trait loci [42,43]. We downloaded the GTEx Analysis V8 eQTL release from the GTEx Portal (https://gtexportal.org). Instrumental SNPs of significant exposures in MR analysis were used to extract significant eQTLs and eQTL nominated genes in obesity-relevant tissues (subcutaneous adipose and visceral adipose). These variant-gene pairs in both tissues were further calculated using the GTEx v8 eQTL Calculator. The expression of eQTL in specific tissues was measured by Normalized effect size (NES).

### Enrichment analysis

In this study, we used the online tool KOBAS-i v3.0 (http://bioinfo.org/kobas/) for functional enrichment analysis of genes identified by eQTL analysis with the KEGG pathway [44]. We analyzed genes expressed in the two types of adipose tissue, respectively. In the set of genes significantly tested after Bonferroni corrections, allelic fold change (aFC) in log2 scale >1 was regarded as an up-regulated gene. otherwise, it was a down-regulated gene. The value was calculated using a hypergeometric distribution and the false discovery rate < 0.05 was considered significant statistically.

### Statistical analysis

All statistical analyses were conducted in R version 4.2.3, utilizing the R package Two Sample MR (version 0.5.7). We applied a Bonferroni correction factor of 10 in our statistical analysis, corresponding to the 10 distinct exposures investigated. This approach, considering the multiple independent analyses performed, led us to set a significance threshold at *p*<0.005 (0.05/10), ensuring a more robust and reliable interpretation of our results.

### Ethics statement

All the data used in this study were sourced from publicly available online databases. As such, participants’ written informed consent had been previously obtained.

## Results

**Fig 3:**
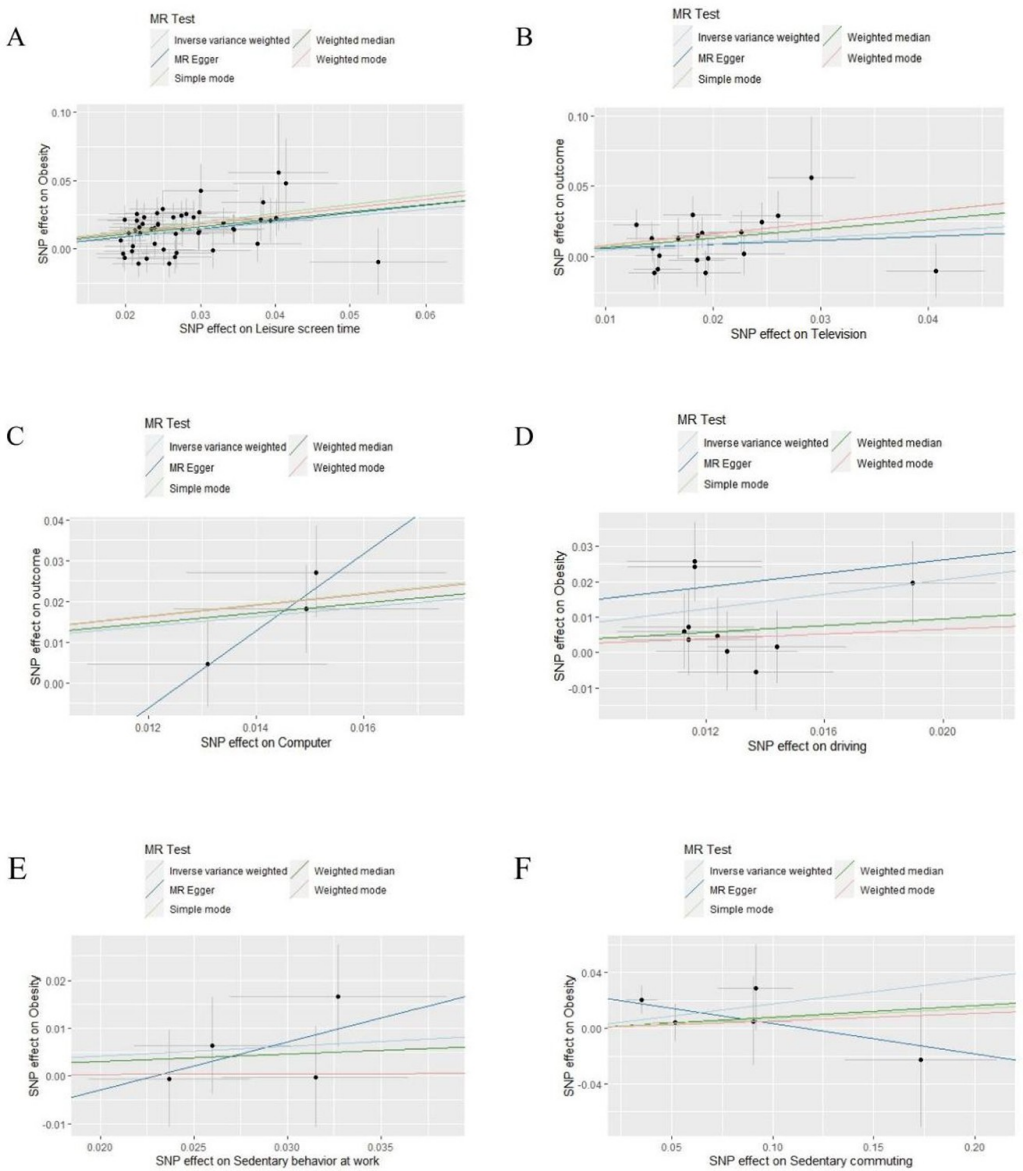
Sensitivity analyses of MR results about SB to Obesity. A: Scatter plots show genetically predicted of Leisure screen time (LST); B: Scatter plots show genetically predicted of Television watching; C: Scatter plots show genetically predicted of Computer use; D: Scatter plots show genetically predicted of Driving on Obesity; E: Scatter plots show genetically predicted of Sedentary behavior at work (SB at work); F: Scatter plots show genetically predicted of sedentary commuting. Note: The term ‘genetically predicted’ refers to the application of genetic methods for predicting individuals’ inclinations towared specific behaviors, leveraging genetic information.

The MR analysis used the Inverse Variance Weighted (IVW) method and revealed, causal relationships between obesity and four genetically predicted specific types of SB: LST (Odds ratio [OR] = 1.62, [95% CI] = [1.43, 1.84], *p* = 6.49×10^−14^), television watching ([OR] = 1.55, [95% CI] = [1.27, 1.90], *p* = 1.67×10^−5^), computer use ([OR] = 1.52, [95% CI] = [1.08, 2.13], *p* = 1.61×10^−2^), and driving ([OR] = 2.79, [95% CI] = [1.25, 6.21], *p* = 1.23×10^−02^). However, no causal relationships were observed with SB at work or sedentary commuting. The detailed findings are depicted in Table 2 and Fig 3. The forest plot and leave-one-out analysis (LOO) plot results are listed in S3 Fig in S1 File.

**Table 2:** MR estimates of the causal association between SB/PA/SD and the risk of obesity.

	Obesity					
Exposure	Methods		OR (95% CI)		*p*	*F-statistic*
LST		MR Egger		1.79 (0.99, 3.21)	0.06	113.19
LST		Weighted median		1.72 (1.44, 2.05)	1.58×10^−9^	
LST		IVW		1.62 (1.43, 1.84)	6.49×10^−14^	
LST		Simple mode		1.91 (1.33, 2.74)	9.47×10^−04^	
LST		Weighted mode		1.83 (1.29, 2.60)	1.50×10^−03^	
Television watching		MR Egger		2.04 (0.92, 4.55)	0.09	47.57
Television watching		Weighted median		1.65 (1.24, 2.20)	5.83×10^−4^	
Television watching		IVW		1.55 (1.27, 1.90)	1.67×10^−5^	
Television watching		Simple mode		2.38 (1.19, 4.74)	0.02	
Television watching		Weighted mode		2.24 (1.21, 4.14)	0.01	
Computer use		MR Egger		27.49 (1.34, 563.20)	0.05	28.11
Computer use		Weighted median		1.60 (1.00, 2.56)	0.05	
Computer use		IVW		1.52 (1.08, 2.13)	1.61×10^−2^	
Computer use		Simple mode		1.61 (0.67, 3.84)	0.30	
Computer use		Weighted mode		1.86 (0.77, 4.46)	0.18	
Driving		MR Egger		2.61 (0.01, 627.55)	0.74	43.88
Driving		Weighted median		1.61 (0.79, 3.27)	0.19	
Driving		IVW		2.79 (1.25, 6.21)	1.23×10^−2^	
Driving		Simple mode		1.39 (0.55, 3.50)	0.50	
Driving		Weighted mode		1.39 (0.55, 3.54)	0.50	
SB at work		MR Egger		2.74 (0.18, 41.59)	0.54	124.45
SB at work		Weighted median		1.17 (0.77, 1.76)	0.46	
SB at work		IVW		1.23 (0.86, 1.75)	0.26	
SB at work		Simple mode		1.01 (0.54, 1.88)	0.97	
SB at work		Weighted mode		1.01 (0.56, 1.84)	0.97	
Sedentary commuting		MR Egger		0.80 (0.46, 1.41)	0.50	53.31
Sedentary commuting		Weighted median		1.09 (0.78, 1.52)	0.63	
Sedentary commuting		IVW		1.19 (0.92, 1.55)	0.18	
Sedentary commuting		Simple mode		1.07 (0.67, 1.73)	0.78	
Sedentary commuting		Weighted mode		1.06 (0.66, 1.69)	0.83	
MVPA		MR Egger		0.08 (0.00, 1.68)	0.65	
MVPA		Weighted median		0.87 (0.44, 1.73)	0.15	30.49
MVPA		IVW		0.88 (0.52, 1.48)	0.70	
MVPA		Simple mode		1.50 (0.44, 5.10)	0.63	
MVPA		Weighted mode		0.64 (0.18, 2.19)	0.53	
Acc425		MR Egger		1.43 (0.56, 3.70)	0.48	17.78
Acc425		Weighted median		0.82 (0.53, 1.27)	0.38	
Acc425		IVW		0.73 (0.52, 1.01)	0.05	
Acc425		Simple mode		0.79 (0.39, 1.60)	0.53	
Acc425		Weighted mode		0.80 (0.41, 1.58)	0.54	
AccAve		MR Egger		0.96 (0.37, 2.49)	0.94	1979.98
AccAve		Weighted median		0.94 (0.88, 1.01)	0.12	
AccAve		IVW		0.96 (0.83, 1.10)	0.53	
AccAve		Simple mode		0.91 (0.81, 1.02)	0.20	
AccAve		Weighted mode		0.92 (0.83, 1.01)	0.19	
SD		MR Egger		0.83 (0.43, 1.57)	0.58	40.65
SD		Weighted median		0.96 (0.71, 1.29)	0.78	
SD		IVW		0.94 (0.76, 1.17)	0.60	
SD		Simple mode		0.90 (0.53, 1.55)	0.72	
SD		Weighted mode		0.82 (0.49, 1.38)	0.48	

Abbreviations: SB, sedentary behavior; PA, physical activity; SD, sleep duration; CI, confidence interval; MR, Mendelian randomization; OR, odds ratio; IVW, Inverse variance weighted; LST, Leisure screen time; SB at work, Sedentary Behavior at work; MVPA, moderate to vigorous physical activity; Acc425, accelerometer-based physical activity with accelerations>425 milli-gravities; AccAve, accelerometer-based physical activity with average acceleration; SD, Sleep duration.

We found no evidence for the causality of genetically predicted relationships among the three indicators: PA, SD, and obesity (MVPA: IVW: OR = 0.88, [95% CI] = [0.52–1.48], *p* = 0.70; Acc425: OR = 0.73, [95% CI] = [0.52–1.01], *p* = 0.05; AccAve: IVW: OR = 0.96, [95% CI] = [0.83–1.10], p = 0.53; SD: IVW: OR = 0.94, [95% CI] = [0.76–1.17], *p* = 0.60)). The weighted median and MR-Egger are listed in Table 2.

### Sensitivity analyses

The MR-Egger intercept and MR-Radial analyses indicated no evidence of pleiotropy in the relationship between PA/SB/SD and obesity. These results strengthen our confidence in drawing robust conclusions from our findings (Table 3). Furthermore, the MR-Egger intercepts for all other results were more significant than *p*>0.05, indicating the absence of horizontal pleiotropy (Table 3). However, we did identify heterogeneity in the results when examining the relationship between AccAve and obesity, as indicated by the Cochran Q test (Q = 22.28, *p* = 5.70×10^−05^). The MR-Radial global test identified that the SNPs rs34517439, rs9293503, and rs62443625 were biased in the results. We conducted further analysis by eliminating these biased SNPs and re-performing MR analysis and sensitivity analysis for AccAve and obesity. This time, the Cochran Q test (*p* = 0.05) and the MR-Radial global test (*p* = 0.54) showed no evidence of heterogeneity in the results, confirming the robustness of the findings.

**Table 3:** Sensitivity analysis of the causal association between PA/SB/SD and the risk of obesity.

Exposure	Outcome	MR-Egger		Cochran Q test	
		Intercept	*p*	Q_value	*p*
LST	Obesity	0.004	0.65	57.67	0.12
Television watching	Obesity	0.003	0.79	59.66	0.22
Computer use	Obesity	-0.12	0.44	16.91	0.53
Driving	Obesity	0.01	0.76	9.08	0.43
Sedentary commuting	Obesity	0.01	0.52	1.93	0.86
SB at work	Obesity	0.01	0.52	1.93	0.86
MVPA	Obesity	0.04	0.17	3.95	0.68
Acc425	Obesity	-0.02	0.17	9.47	0.39
AccAve	Obesity	-0.001	1.00	3.94	0.05
SD	Obesity	0.01	0.68	7.61	0.37

Abbreviations: LST, Leisure screen time; SB at work, Sedentary Behavior at work; MVPA, moderate to vigorous physical activity; Acc425, accelerometer-based physical activity with accelerations >425 milli-gravities; AccAve, accelerometer-based physical activity with average acceleration; SD, Sleep duration.

### eQTL analysis

In our comprehensive study of subcutaneous and visceral adipose tissues, we identified a substantial number of eQTLs associated with prolonged SB, TV watching, computer use, driving, and LST. Specifically, we found 214 eQTLs related to TV watching, 79 related to computer use, 14 related to driving, and 139 related to LST.

The Adipose-subcutaneous tissue found a strong association between the gene *RPS26* and the rs10876864 with TV watching (*p* = 1.4×10–233, NES = -1.2) (*p* = 1.4×10^−233^, NES = -1.2). This association was also evident in the visceral adipose tissue (*p* = 3.4×10^−175^, NES = -1.2). For eQTLs associated with computer usage, the gene *TTC12* and rs2734849 in the subcutaneous adipose tissue demonstrated a noteworthy connection (*p* = 1.9×10^−48^, NES = -0.63). In the context of driving, the visceral adipose tissue exhibited a significant association between the gene *CCDC92* and rs4765541 (*p* = 3.6×10^−27^, NES = 0.34), in the eQTLs related to LST, a prominent association was observed between the gene *NICN1* and rs7615206 in the subcutaneous adipose tissue (*p* = 5.80×10^−4^, NES = -0.09).

### Enrichment analysis

In the gene upregulation KEGG enrichment analysis, we found that these genes are mainly involved in multiple biological pathways. These include the synthesis and secretion of cortisol, thyroid hormone synthesis, insulin secretion, and the longevity-regulating pathway, which showed the most significant enrichment. The analysis also highlighted other pathways such as the synthesis and secretion of aldosterone, the synthesis, secretion, and action of parathyroid hormone, and lysosomal pathways, as detailed in S4 Fig in S1 File and S2 Table in S2 File.

## Discussion

Our study provided genetic evidence supporting the causal relationship between obesity and four types of SB: television watching, LST, computer use and driving. No causal relationships were observed between SB at work, sedentary commuting, PA, SD, and obesity. These findings provided genetic insights into the association of complex traits of SB with obesity. Sensitivity analyses indicated no heterogeneity, horizontal pleiotropy, or influential SNPs identified through the LOO analysis, which further strengthens the reliability and robustness of the results.

Previous observational studies have shown a correlation between SB and obesity [45,46], with dose-response studies indicating a nonlinear association [21]. Furthermore, an observational study involving European and American adults revealed that sitting time is associated with obesity, independent of PA [47,48]. All the studies mentioned above only provided evidence to arouse awareness of SB’s possible impact on obesity. Despite the risk of reverse causality in observational studies, our cost-effective MR approach identified significant causal effects of most types of SB on obesity development, excluding those related to work and commuting. These findings are consistent with prior research on television watching and computer use [21,49]. Additionally, a recent GWAS highlighted the significant impact of LST on obesity, suggesting it is much greater than the reverse [35]. Nevertheless, our study offers solid evidence of a causal relationship between specific types of SB and obesity, contributing significantly to research in this field. It is also crucial to acknowledge that certain types of SB, such as SB at work, did not demonstrate a causal association with obesity, which is an important consideration for future research and public health strategies.

The association between SB and obesity is influenced by direct physiological mechanisms and complex genetic factors. SB reduces muscle activity [50,51], leading to decreased lipoprotein lipase activity essential for lipid metabolism, contributing to fat accumulation and potential obesity [50–52]. It is also associated with higher postprandial glucose and lipid levels, raising the risk of obesity [53,54]. Our study reveals that various SBs, such as TV watching, computer use, driving, and LST, have a significant genetic component, as evidenced by our eQTL analysis in both subcutaneous and visceral adipose tissues. The identification of numerous behavior-specific eQTLs highlights a complex interaction between genetics and lifestyle. For example, the association between the RPS26 gene and the rs10876864 in relation to TV watching, observed in both types of adipose tissues, suggests a genetic inclination towards the effects of extended visual media consumption on fat tissue function. Associations with other genes like TTC12, CCDC92, and NICN1 in contexts of computer use, driving, and LST further support this. Additionally, our study explores the impact of SB on distinct biological processes in various fat tissues, particularly in hormone secretion pathways vital for endocrine regulation. A key finding is the pronounced gene enrichment related to cortisol synthesis and secretion in visceral adipose tissue, indicating that SB might modulate cortisol levels, thus affecting the balance between visceral fat and metabolic health. This aligns with the Rodriguez et al study [55] indicating that cortisol release and fat accumulation are aberrantly regulated in obesity. Furthermore, SB’s potential impact on thyroid hormone synthesis, energy metabolism, and body temperature regulation is notable. The observed gene enrichment related to insulin secretion in visceral fat tissues implies that SB might indirectly heighten the risk of metabolic diseases like diabetes, a significance also emphasized in the study by Chen et al [56]. This comprehensive analysis enhances our understanding of the complex genetic underpinnings, biological mechanisms, and their association with obesity.

In the field of obesity research, various scholars have presented differing viewpoints. Some observational studies [15,53] have found that increasing PA can reduce the risk of obesity and that there is a longitudinal correlation between SD and obesity [53]. However, other studies, such as those by Song et al. [57] and Bell et al. [22], have pointed out that the relationship between PA, SD, and obesity is not clear and remains controversial. Our MR results indicated that PA and longer SD are associated with a decreased trend in obesity risk. These results align with some observational studies [15,22] suggesting a potential protective effect of PA and SD against obesity. However, these associations are not sufficient to prove a causal relationship. Observational studies [57] indicate that obesity is influenced by interactions between SB, PA, and genetic factors like the MC4R gene variant, without PA alone showing a direct causal effect [22]. Additionally, both prolonged and short SD, along with lower sleep satisfaction, are linked to increased obesity risk across all ages [1,25], potentially due to their impact on appetite control, which can lead to higher food intake and reduced energy expenditure [6]. However, the precise biological mechanisms between sleep and obesity remain unclear. Therefore, although previous literature provides evidence suggesting that PA and SD might be beneficial in resisting obesity, our study results indicate that there is no direct causal association between these factors and obesity risk. To more comprehensively understand the impact of PA and SD on obesity risk, it is necessary to delve deeper into the biological mechanisms between them. This may include studying changes in sleep patterns, appetite regulation, energy metabolism, and how these changes affect the development of obesity. By gaining a deeper understanding of these biological processes, we can better comprehend how PA and SD influence the risk of obesity and provide a scientific basis for future prevention and treatment strategies.

Historically, while interventions focused on PA and sleep or diet [23], the role of SB in obesity was neglected, and traditional calorie-focused approaches have not consistently achieved long-term weight control success [54]. Through our exploration into the causal relationships between SB, PA, SD and obesity, we identified distinct causal associations between four SBs and obesity. Notably, we found significant positive associations between these four SBs and the risk of obesity. These findings challenge previous obesity strategies and suggest that prolonged sitting should be a focal point for interventions targeting people with obesity. However, further randomized controlled trials (RCTs) are recommended to confirm its effectiveness.

Our study has several strengths. First, we comprehensively analyzed six types of SB in GWAS data and explored the biological mechanisms of how SB contributes to its development, which allowed us to gain deeper insights into the diverse impacts of different SBs on obesity. Second, we minimized potential bias from different racial backgrounds by focusing on individuals with European ancestry. Third, we used separate GWAS and Finnish databases to select exposure and outcome data to avoid overlapping samples. Fourth, we conducted multiple sensitivity analyses to assess the validity of MR assumptions and applied MR-radial to minimize heterogeneity to the greatest extent possible. However, our study also has limitations. First, its findings primarily apply to European populations, with limited generalizability due to sparse disease information in GWAS data, hindering stratified analyses. Second, while offering robust evidence, our results necessitate careful interpretation due to possible unmeasured confounders and residual influences from unobserved pleiotropy. Third, the analysis power might be compromised by the small number of SNPs for certain traits, and the scarcity of SNPs for driving could yield imprecise estimates. Finally, multicollinearity among phenotypes such as LST, watching television, and computer use may obscure the ability to isolate their independent impacts.

## Conclusion

Our study underscores the importance of adopting an active lifestyle and reducing SB to combat the risk of obesity effectively. Compared to encouraging regular PA and SD, reducing specific SBs (especially LST, television watching, computer use, and driving) could be a more targeted and practical approach to combat obesity. Additionally, our analysis suggests genetic associations and biological pathways related to obesity, enhancing our understanding of its mechanisms. Future research should delve deeper into these aspects to devise more targeted and effective obesity prevention and management strategies.

## Supporting information

S1 Checklist STROBE-MR guideline. (DOCX)

S1 File S3 Fig. Forest plot and LOO plot for SBs on obesity. S4 Fig. Result of eQTL enrichment analysis.
(DOCX)

S2 File S1 Table. Instrument variables of LST. S2 Table. Instrument variables of television watching. S3 Table. Instrument variables of computer use. S4 Table. Instrument variables of driving. S5 Table. Instrument variables of SB at work. S6 Table. Instrument variables of sedentary commuting. S7 Table. Instrument variables of MVPA. S8 Table. Instrument variables of AccAve. S9 Table. Instrument variables of Acc425. S10 Table. Instrument variables of SD. S11 Table. Result of eQTL. S12 Table. Result of eQTL enrichment analysis.
(XLSX)

## References

[1] Bacaro V, Ballesio A, Cerolini S, Vacca M, Poggiogalle E, Donini LM, et al. Sleep duration and obesity in adulthood: An updated systematic review and meta-analysis. Obes Res Clin Pract. 2020;14(4):301–9. Epub 2020/06/13. doi: 10.1016/j.orcp.2020.03.004 . 32527625

[2] Popkin BM, Adair LS, Ng SW. Global nutrition transition and the pandemic of obesity in developing countries. Nutr Rev. 2012;70(1):3–21. Epub 2012/01/10. doi: 10.1111/j.1753-4887.2011.00456.x ; PubMed Central PMCID: PMC3257829. 22221213 PMC3257829

[3] Smith KB, Smith MS. Obesity Statistics. Prim Care. 2016;43(1):121–35, ix. Epub 2016/02/21. doi: 10.1016/j.pop.2015.10.001 . 26896205

[4] Collaborators GBDO, Afshin A, Forouzanfar MH, Reitsma MB, Sur P, Estep K, et al. Health Effects of Overweight and Obesity in 195 Countries over 25 Years. N Engl J Med. 2017;377(1):13–27. Epub 2017/06/13. doi: 10.1056/NEJMoa1614362 ; PubMed Central PMCID: PMC5477817. 28604169 PMC5477817

[5] Finkelstein EA, Khavjou OA, Thompson H, Trogdon JG, Pan L, Sherry B, et al. Obesity and severe obesity forecasts through 2030. Am J Prev Med. 2012;42(6):563–70. Epub 2012/05/23. doi: 10.1016/j.amepre.2011.10.026 . 22608371

[6] Ng M, Fleming T, Robinson M, Thomson B, Graetz N, Margono C, et al. Global, regional, and national prevalence of overweight and obesity in children and adults during 1980–2013: a systematic analysis for the Global Burden of Disease Study 2013. Lancet. 2014;384(9945):766–81. Epub 2014/06/02. doi: 10.1016/S0140-6736(14)60460-8 ; PubMed Central PMCID: PMC4624264. 24880830 PMC4624264

[7] Yanovski SZ, Yanovski JA. Toward Precision Approaches for the Prevention and Treatment of Obesity. JAMA. 2018;319(3):223–4. Epub 2018/01/18. doi: 10.1001/jama.2017.20051 ; PubMed Central PMCID: PMC5787370. 29340687 PMC5787370

[8] Bhaskaran K, Douglas I, Forbes H, dos-Santos-Silva I, Leon DA, Smeeth L. Body-mass index and risk of 22 specific cancers: a population-based cohort study of 5.24 million UK adults. Lancet. 2014;384(9945):755–65. Epub 2014/08/19. doi: 10.1016/S0140-6736(14)60892-8 ; PubMed Central PMCID: PMC4151483. 25129328 PMC4151483

[9] Visscher TL, Seidell JC. The public health impact of obesity. Annu Rev Public Health. 2001;22:355–75. Epub 2001/03/29. doi: 10.1146/annurev.publhealth.22.1.355 . 11274526

[10] Gortmaker SL, Must A, Perrin JM, Sobol AM, Dietz WH. Social and economic consequences of overweight in adolescence and young adulthood. N Engl J Med. 1993;329(14):1008–12. Epub 1993/09/30. doi: 10.1056/NEJM199309303291406 . 8366901

[11] Wang YC, McPherson K, Marsh T, Gortmaker SL, Brown M. Health and economic burden of the projected obesity trends in the USA and the UK. Lancet. 2011;378(9793):815–25. Epub 2011/08/30. doi: 10.1016/S0140-6736(11)60814-3 . 21872750

[12] Tremblay MS, Aubert S, Barnes JD, Saunders TJ, Carson V, Latimer-Cheung AE, et al. Sedentary Behavior Research Network (SBRN)—Terminology Consensus Project process and outcome. Int J Behav Nutr Phys Act. 2017;14(1):75. Epub 2017/06/11. doi: 10.1186/s12966-017-0525-8 ; PubMed Central PMCID: PMC5466781. 28599680 PMC5466781

[13] Bluher M. Obesity: global epidemiology and pathogenesis. Nat Rev Endocrinol. 2019;15(5):288–98. Epub 2019/03/01. doi: 10.1038/s41574-019-0176-8 . 30814686

[14] Ekelund U, Tarp J, Fagerland MW, Johannessen JS, Hansen BH, Jefferis BJ, et al. Joint associations of accelero-meter measured physical activity and sedentary time with all-cause mortality: a harmonised meta-analysis in more than 44 000 middle-aged and older individuals. Br J Sports Med. 2020;54(24):1499–506. Epub 2020/11/27. doi: 10.1136/bjsports-2020-103270 ; PubMed Central PMCID: PMC7719907. 33239356 PMC7719907

[15] Pavey TG, Peeters GM, Gomersall SR, Brown WJ. Long-term Effects of Physical Activity Level on Changes in Healthy Body Mass Index Over 12 Years in Young Adult Women. Mayo Clin Proc. 2016;91(6):735–44. Epub 2016/05/05. doi: 10.1016/j.mayocp.2016.03.008 . 27143482

[16] Montgomerie AM, Chittleborough CR, Taylor AW. Physical inactivity and incidence of obesity among South Australian adults. PloS one. 2014;9(11):e112693. Epub 2014/11/11. doi: 10.1371/journal.pone.0112693 ; PubMed Central PMCID: PMC4226631. 25383626 PMC4226631

[17] Kim J. Sleep Duration and Obesity. J Obes Metab Syndr. 2017;26(1):1–2. Epub 2017/03/01. doi: 10.7570/jomes.2017.26.1.1 ; PubMed Central PMCID: PMC6484924. 31089486 PMC6484924

[18] Sunwoo JS, Yang KI, Kim JH, Koo DL, Kim D, Hong SB. Sleep duration rather than sleep timing is associated with obesity in adolescents. Sleep Med. 2020;68:184–9. Epub 2020/02/12. doi: 10.1016/j.sleep.2019.12.014 . 32044556

[19] Rosenberger ME, Fulton JE, Buman MP, Troiano RP, Grandner MA, Buchner DM, et al. The 24-Hour Activity Cycle: A New Paradigm for Physical Activity. Med Sci Sports Exerc. 2019;51(3):454–64. Epub 2018/10/20. doi: 10.1249/MSS.0000000000001811 ; PubMed Central PMCID: PMC6377291. 30339658 PMC6377291

[20] Silveira EA, Mendonca CR, Delpino FM, Elias Souza GV, Pereira de Souza Rosa L, de Oliveira C, et al. Sedentary behavior, physical inactivity, abdominal obesity and obesity in adults and older adults: A systematic review and meta-analysis. Clin Nutr ESPEN. 2022;50:63–73. Epub 2022/07/26. doi: 10.1016/j.clnesp.2022.06.001 . 35871953

[21] Guo C, Zhou Q, Zhang D, Qin P, Li Q, Tian G, et al. Association of total sedentary behaviour and television viewing with risk of overweight/obesity, type 2 diabetes and hypertension: A dose-response meta-analysis. Diabetes Obes Metab. 2020;22(1):79–90. Epub 2019/08/31. doi: 10.1111/dom.13867 . 31468597

[22] Bell JA, Hamer M, Batty GD, Singh-Manoux A, Sabia S, Kivimaki M. Combined effect of physical activity and leisure time sitting on long-term risk of incident obesity and metabolic risk factor clustering. Diabetologia. 2014;57(10):2048–56. Epub 2014/08/01. doi: 10.1007/s00125-014-3323-8 ; PubMed Central PMCID: PMC4153972. 25078481 PMC4153972

[23] Foster-Schubert KE, Alfano CM, Duggan CR, Xiao L, Campbell KL, Kong A, et al. Effect of diet and exercise, alone or combined, on weight and body composition in overweight-to-obese postmenopausal women. Obesity (Silver Spring). 2012;20(8):1628–38. Epub 2011/04/16. doi: 10.1038/oby.2011.76 ; PubMed Central PMCID: PMC3406229. 21494229 PMC3406229

[24] Cappuccio FP, Taggart FM, Kandala NB, Currie A, Peile E, Stranges S, et al. Meta-analysis of short sleep duration and obesity in children and adults. Sleep. 2008;31(5):619–26. Epub 2008/06/04. doi: 10.1093/sleep/31.5.619 ; PubMed Central PMCID: PMC2398753. 18517032 PMC2398753

[25] Norton MC, Eleuteri S, Cerolini S, Ballesio A, Conte SC, Falaschi P, et al. Is poor sleep associated with obesity in older adults? A narrative review of the literature. Eat Weight Disord. 2018;23(1):23–38. Epub 2017/10/31. doi: 10.1007/s40519-017-0453-2 . 29080950

[26] Banks E, Lim L, Seubsman SA, Bain C, Sleigh A. Relationship of obesity to physical activity, domestic activities, and sedentary behaviours: cross-sectional findings from a national cohort of over 70,000 Thai adults. BMC Public Health. 2011;11:762. Epub 2011/10/06. doi: 10.1186/1471-2458-11-762 ; PubMed Central PMCID: PMC3204261. 21970620 PMC3204261

[27] Mitchell JA, Bottai M, Park Y, Marshall SJ, Moore SC, Matthews CE. A prospective study of sedentary behavior and changes in the body mass index distribution. Med Sci Sports Exerc. 2014;46(12):2244–52. Epub 2014/05/02. doi: 10.1249/MSS.0000000000000366 ; PubMed Central PMCID: PMC4211994. 24781893 PMC4211994

[28] Pedisic Z, Grunseit A, Ding D, Chau JY, Banks E, Stamatakis E, et al. High sitting time or obesity: Which came first? Bidirectional association in a longitudinal study of 31,787 Australian adults. Obesity (Silver Spring). 2014;22(10):2126–30. Epub 2014/06/20. doi: 10.1002/oby.20817 ; PubMed Central PMCID: PMC4265269. 24943057 PMC4265269

[29] Gala H, Tomlinson I. The use of Mendelian randomisation to identify causal cancer risk factors: promise and limitations. J Pathol. 2020;250(5):541–54. Epub 2020/03/11. doi: 10.1002/path.5421 . 32154591

[30] Burgess S, Thompson SG. Multivariable Mendelian randomization: the use of pleiotropic genetic variants to estimate causal effects. Am J Epidemiol. 2015;181(4):251–60. Epub 2015/01/30. doi: 10.1093/aje/kwu283 ; PubMed Central PMCID: PMC4325677. 25632051 PMC4325677

[31] Davey Smith G, Hemani G. Mendelian randomization: genetic anchors for causal inference in epidemiological studies. Hum Mol Genet. 2014;23(R1):R89–98. Epub 2014/07/30. doi: 10.1093/hmg/ddu328 ; PubMed Central PMCID: PMC4170722. 25064373 PMC4170722

[32] Cornish AJ, Tomlinson IPM, Houlston RS. Mendelian randomisation: A powerful and inexpensive method for identifying and excluding non-genetic risk factors for colorectal cancer. Mol Aspects Med. 2019;69:41–7. Epub 2019/02/03. doi: 10.1016/j.mam.2019.01.002 ; PubMed Central PMCID: PMC6856712. 30710596 PMC6856712

[33] Skrivankova VW, Richmond RC, Woolf BAR, Davies NM, Swanson SA, VanderWeele TJ, et al. Strengthening the reporting of observational studies in epidemiology using mendelian randomisation (STROBE-MR): explanation and elaboration. BMJ. 2021;375:n2233. Epub 2021/10/28. doi: 10.1136/bmj.n2233 ; PubMed Central PMCID: PMC8546498 at www.icmje.org/disclosure-of-interest/ and declare: support from the SNSF, NIHR Biomedical Research Centre at University Hospitals Bristol, Weston NHS Foundation Trust, and University of Bristol for the submitted work; no financial relationships with any organisations that might have an interest in the submitted work in the previous three years; EWL (head of research at The BMJ) played no part in the peer review or decision making of this paper at the editorial level, and contributed solely as an author; no other relationships or activities that could appear to have influenced the submitted work. Provenance and peer review: Not commissioned; externally peer reviewed. 34702754 PMC8546498

[34] van de Vegte YJ, Said MA, Rienstra M, van der Harst P, Verweij N. Genome-wide association studies and Mendelian randomization analyses for leisure sedentary behaviours. Nature communications. 2020;11(1):1770. Epub 2020/04/23. doi: 10.1038/s41467-020-15553-w ; PubMed Central PMCID: PMC7174427. 32317632 PMC7174427

[35] Wang Z, Emmerich A, Pillon NJ, Moore T, Hemerich D, Cornelis MC, et al. Genome-wide association analyses of physical activity and sedentary behavior provide insights into underlying mechanisms and roles in disease prevention. Nat Genet. 2022;54(9):1332–44. Epub 2022/09/08. doi: 10.1038/s41588-022-01165-1 ; PubMed Central PMCID: PMC9470530. 36071172 PMC9470530

[36] Doherty A, Smith-Byrne K, Ferreira T, Holmes MV, Holmes C, Pulit SL, et al. GWAS identifies 14 loci for device-measured physical activity and sleep duration. Nat Commun. 2018;9(1):5257. Epub 2018/12/12. doi: 10.1038/s41467-018-07743-4 ; PubMed Central PMCID: PMC6288145. 30531941 PMC6288145

[37] Aziz M, Ali SS, Das S, Younus A, Malik R, Latif MA, et al. Association of Subjective and Objective Sleep Duration as well as Sleep Quality with Non-Invasive Markers of Sub-Clinical Cardiovascular Disease (CVD): A Systematic Review. J Atheroscler Thromb. 2017;24(3):208–26. Epub 2016/11/15. doi: 10.5551/jat.36194 ; PubMed Central PMCID: PMC5383537. 27840384 PMC5383537

[38] Carrasquilla GD, Garcia-Urena M, Fall T, Sorensen TIA, Kilpelainen TO. Mendelian randomization suggests a bidirectional, causal relationship between physical inactivity and adiposity. Elife. 2022;11. Epub 2022/03/08. doi: 10.7554/eLife.70386 ; PubMed Central PMCID: PMC8975550. 35254260 PMC8975550

[39] Bowden J, Davey Smith G, Burgess S. Mendelian randomization with invalid instruments: effect estimation and bias detection through Egger regression. Int J Epidemiol. 2015;44(2):512–25. Epub 2015/06/08. doi: 10.1093/ije/dyv080 ; PubMed Central PMCID: PMC4469799. 26050253 PMC4469799

[40] Zhang M, Wang W, Li M, Sheng H, Zhai Y. Efficacy of Mobile Health Applications to Improve Physical Activity and Sedentary Behavior: A Systematic Review and Meta-Analysis for Physically Inactive Individuals. Int J Environ Res Public Health. 2022;19(8). Epub 2022/04/24. doi: 10.3390/ijerph19084905 ; PubMed Central PMCID: PMC9031730. 35457775 PMC9031730

[41] Zou M, Zhang W, Shen L, Xu Y, Zhu Y. Causal association between inflammatory bowel disease and herpes virus infections: a two-sample bidirectional Mendelian randomization study. Front Immunol. 2023;14:1203707. Epub 2023/07/19. doi: 10.3389/fimmu.2023.1203707 ; PubMed Central PMCID: PMC10351388. 37465669 PMC10351388

[42] Consortium GT. Human genomics. The Genotype-Tissue Expression (GTEx) pilot analysis: multitissue gene regulation in humans. Science. 2015;348(6235):648–60. Epub 2015/05/09. doi: 10.1126/science.1262110 ; PubMed Central PMCID: PMC4547484. 25954001 PMC4547484

[43] Li J, Huang T. Predicting and analyzing early wake-up associated gene expressions by integrating GWAS and eQTL studies. Biochim Biophys Acta Mol Basis Dis. 2018;1864(6 Pt B):2241–6. Epub 2017/11/08. doi: 10.1016/j.bbadis.2017.10.036 . 29109033

[44] Bu D, Luo H, Huo P, Wang Z, Zhang S, He Z, et al. KOBAS-i: intelligent prioritization and exploratory visualization of biological functions for gene enrichment analysis. Nucleic Acids Res. 2021;49(W1):W317–W25. Epub 2021/06/05. doi: 10.1093/nar/gkab447 ; PubMed Central PMCID: PMC8265193. 34086934 PMC8265193

[45] Hamilton MT. The role of skeletal muscle contractile duration throughout the whole day: reducing sedentary time and promoting universal physical activity in all people. J Physiol. 2018;596(8):1331–40. Epub 2017/06/29. doi: 10.1113/JP273284 ; PubMed Central PMCID: PMC5899982. 28657123 PMC5899982

[46] Bey L, Hamilton MT. Suppression of skeletal muscle lipoprotein lipase activity during physical inactivity: a molecular reason to maintain daily low-intensity activity. J Physiol. 2003;551(Pt 2):673–82. Epub 2003/06/20. doi: 10.1113/jphysiol.2003.045591 ; PubMed Central PMCID: PMC2343229. 12815182 PMC2343229

[47] O’Keefe JH, Bell DS. Postprandial hyperglycemia/hyperlipidemia (postprandial dysmetabolism) is a cardiovascular risk factor. Am J Cardiol. 2007;100(5):899–904. Epub 2007/08/28. doi: 10.1016/j.amjcard.2007.03.107 . 17719342

[48] Hamilton MT, Hamilton DG, Zderic TW. Role of low energy expenditure and sitting in obesity, metabolic syndrome, type 2 diabetes, and cardiovascular disease. Diabetes. 2007;56(11):2655–67. Epub 2007/09/11. doi: 10.2337/db07-0882 . 17827399

[49] Shields M, Tremblay MS. Sedentary behaviour and obesity. Health Rep. 2008;19(2):19–30. Epub 2008/07/23. . 18642516

[50] Bowman SA. Television-viewing characteristics of adults: correlations to eating practices and overweight and health status. Prev Chronic Dis. 2006;3(2):A38. Epub 2006/03/17. ; PubMed Central PMCID: PMC1563980. 16539779 PMC1563980

[51] Harris JL, Bargh JA, Brownell KD. Priming effects of television food advertising on eating behavior. Health Psychol. 2009;28(4):404–13. Epub 2009/07/15. doi: 10.1037/a0014399 ; PubMed Central PMCID: PMC2743554. 19594263 PMC2743554

[52] Benatti FB, Ried-Larsen M. The Effects of Breaking up Prolonged Sitting Time: A Review of Experimental Studies. Med Sci Sports Exerc. 2015;47(10):2053–61. Epub 2015/09/18. doi: 10.1249/MSS.0000000000000654 . 26378942

[53] Liu W, Zhang R, Tan A, Ye B, Zhang X, Wang Y, et al. Long sleep duration predicts a higher risk of obesity in adults: a meta-analysis of prospective cohort studies. J Public Health (Oxf). 2019;41(2):e158–e68. Epub 2018/08/15. doi: 10.1093/pubmed/fdy135 . 30107483

[54] Mathis BJ, Tanaka K, Hiramatsu Y. Metabolically Healthy Obesity: Are Interventions Useful? Curr Obes Rep. 2023;12(1):36–60. Epub 2023/02/23. doi: 10.1007/s13679-023-00494-4 . 36814043

[55] Incollingo Rodriguez AC, Epel ES, White ML, Standen EC, Seckl JR, Tomiyama AJ. Hypothalamic-pituitary-adrenal axis dysregulation and cortisol activity in obesity: A systematic review. Psychoneuroendocrinology. 2015;62:301–18. Epub 2015/09/12. doi: 10.1016/j.psyneuen.2015.08.014 . 26356039

[56] Chen L, Zhang YH, Li J, Wang S, Zhang Y, Huang T, et al. Deciphering the Relationship between Obesity and Various Diseases from a Network Perspective. Genes. 2017;8(12). Epub 2017/12/21. doi: 10.3390/genes8120392 ; PubMed Central PMCID: PMC5748710. 29258237 PMC5748710

[57] Song JY, Song QY, Wang S, Ma J, Wang HJ. Physical Activity and Sedentary Behaviors Modify the Association between Melanocortin 4 Receptor Gene Variant and Obesity in Chinese Children and Adolescents. PloS one. 2017;12(1):e0170062. Epub 2017/01/13. doi: 10.1371/journal.pone.0170062 ; PubMed Central PMCID: PMC5231371. 28081251 PMC5231371

