# Correction: Sedentary behavior, physical activity, sleep duration and obesity risk: Mendelian randomization study

**DOI:** 10.1371/journal.pone.0315961

**Published:** 2024-12-12

**Authors:** Siqing Chen, Lili Yang, Yuting Yang, Wenmini Shi, Matthew Stults-Kolehmainen, Qiao Yuan, Chenchen Wang, Jing Ye

There are errors in the author affiliations. The correct affiliations are as follows:

Siqing Chen^1,2^, Lili Yang^1^, Yuting Yang^1^, Wenmini Shi^3^, Matthew StultsKolehmainen^2,4^, Qiao Yuan^1^, Chenchen Wang^1^, Jing Ye^1^

**1** Department of Nursing, the Fourth Affiliated Hospital of School of Medicine, and International School of Medicine, International Institutes of Medicine, Zhejiang University, Yiwu, China, **2** Department of Biobehavioral Sciences, Teachers College-Columbia University, New York, NY, United States of America, **3** Li Ka Shing Faculty of Medicine, School of Public Health, The University of Hong Kong, Hong Kong, SAR China, **4** Center for Weight Management, Yale New Haven Hospital, New Haven, CT, United States.
