# Supplementary material for: Sedentary behavior, physical activity, sleep duration and obesity risk: Mendelian randomization study
Source: PLoS One. 2024 Mar 8;19(3):e0300074. doi: 10.1371/journal.pone.0300074 (PMC10923474; doi:10.1371/journal.pone.0300074)
Supplement: S1 File — S4 Fig. Result of eQTL enrichment analysis. (DOCX) [file pone.0300074.s002.docx]

**Sedentary behavior, physical activity, sleep duration and obesity risk: Mendelian randomization study**

Siqing Chen^1^, Lili Yang^1*^, Yuting Yang^1^, Wenming Shi^2^, [Matthew Stults-Kolehmainen](https://www.researchgate.net/profile/Matthew-Stults-Kolehmainen)^3,4^, Qiao Yuan^1^, Chenchen Wang^1^, Jing Ye^1^

Additional file 2: Figures

A
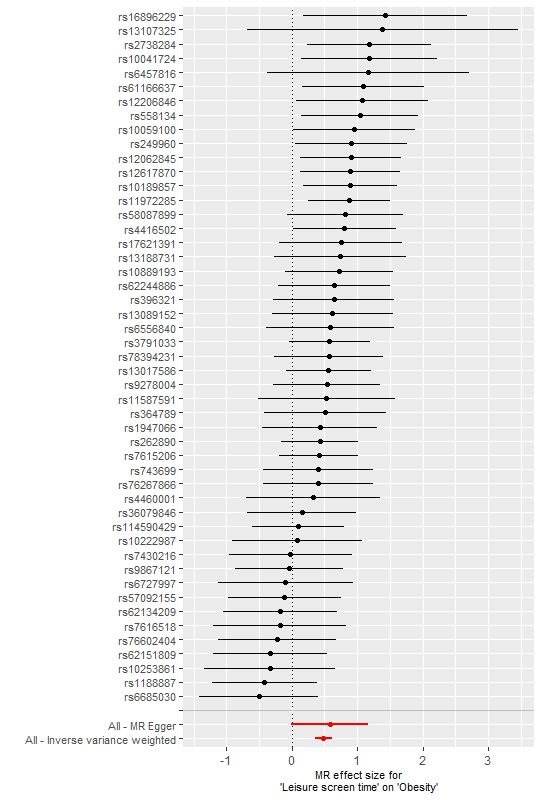
 B
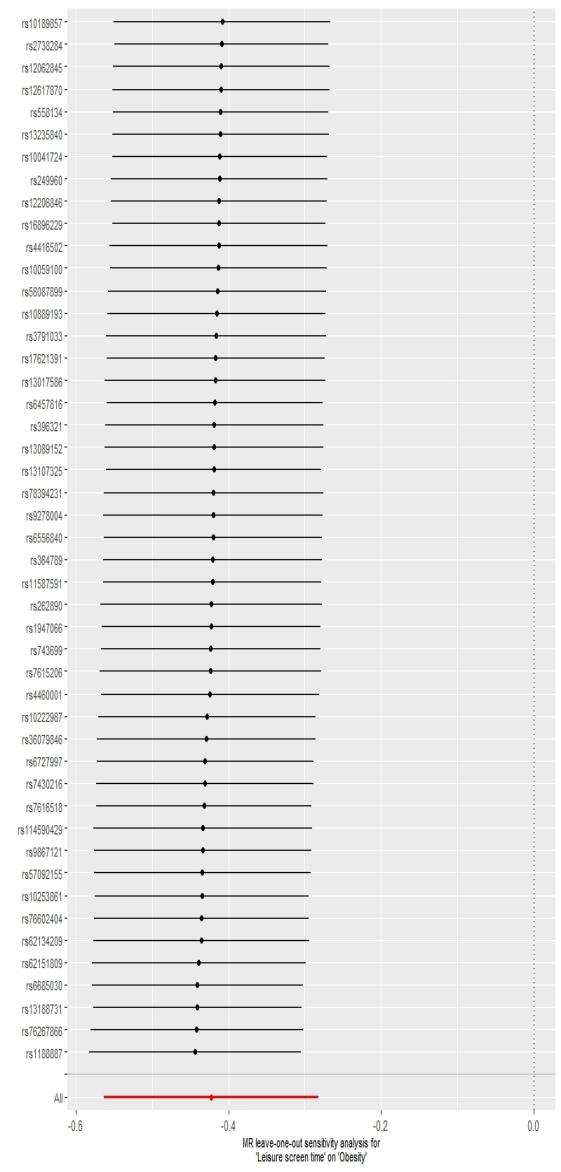


Fig S1. forest plot(A) and leave-one-out analysis(B) for LST on obesity.


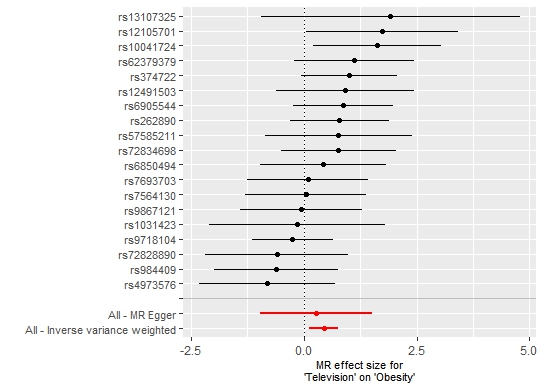


B
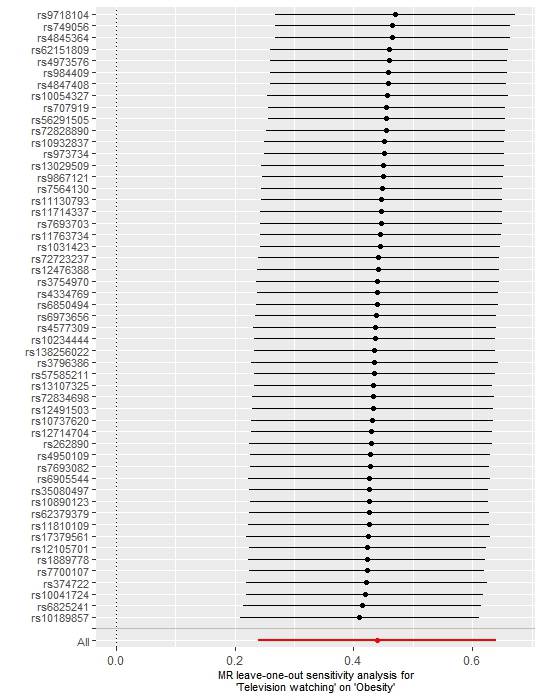


Fig S2. forest plot(A) and leave-one-out analysis(B) for television watching on obesity.

A
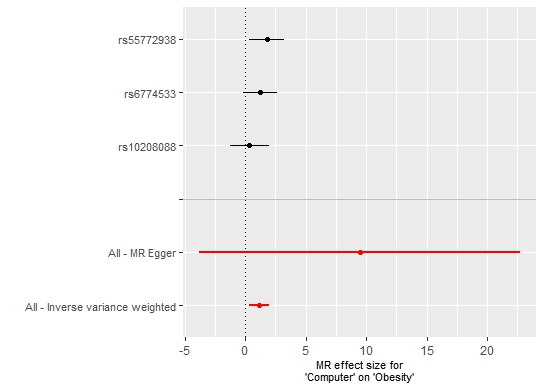


B
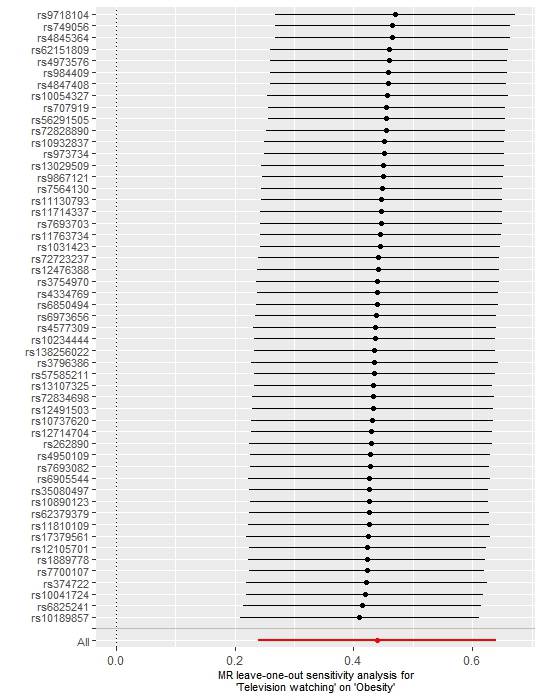


Fig S3. forest plot(A) and leave-one-out analysis(B) for computer use on obesity.

A
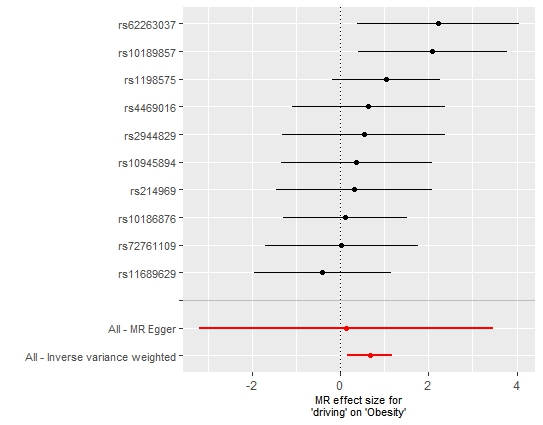


B
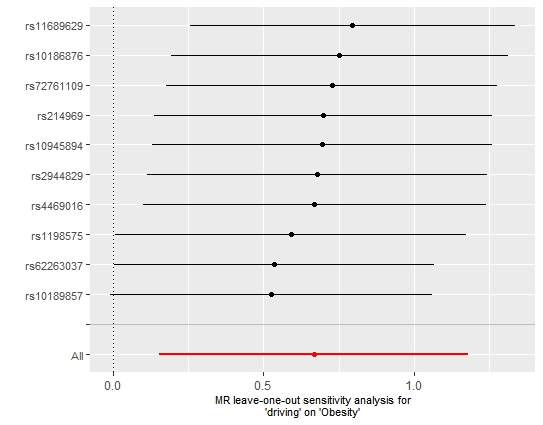


Fig S4. forest plot(A) and leave-one-out analysis(B) for driving on obesity.

A
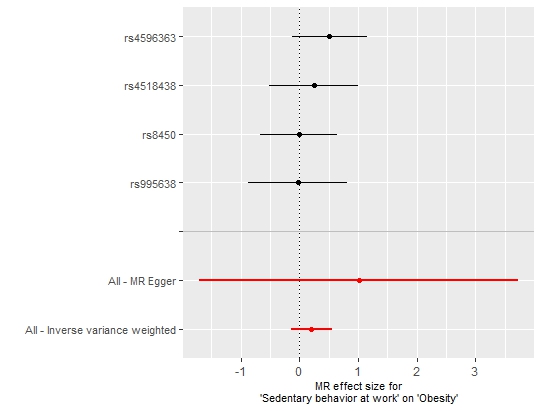


B
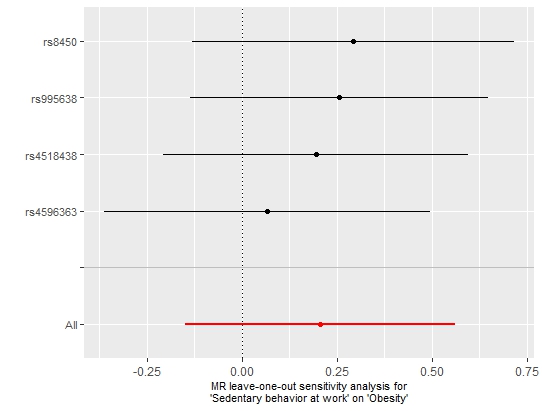


Fig S5. forest plot(A) and leave-one-out analysis(B) for SB at work on obesity.

A
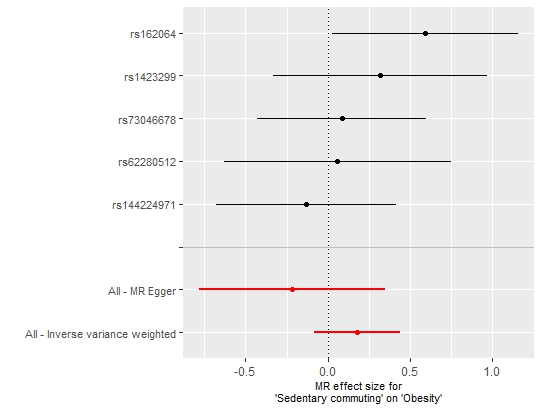


B
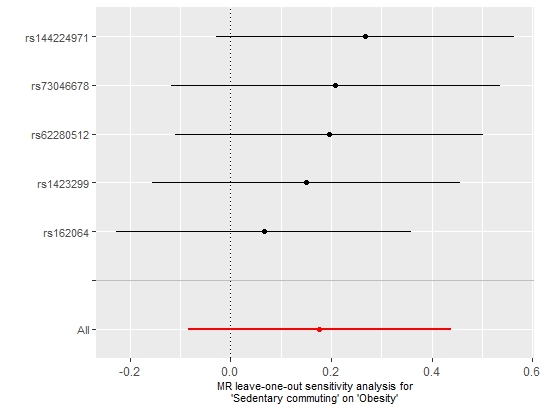


Fig S6. forest plot(A) and leave-one-out analysis(B) for Sedentary commuting on Obesity.


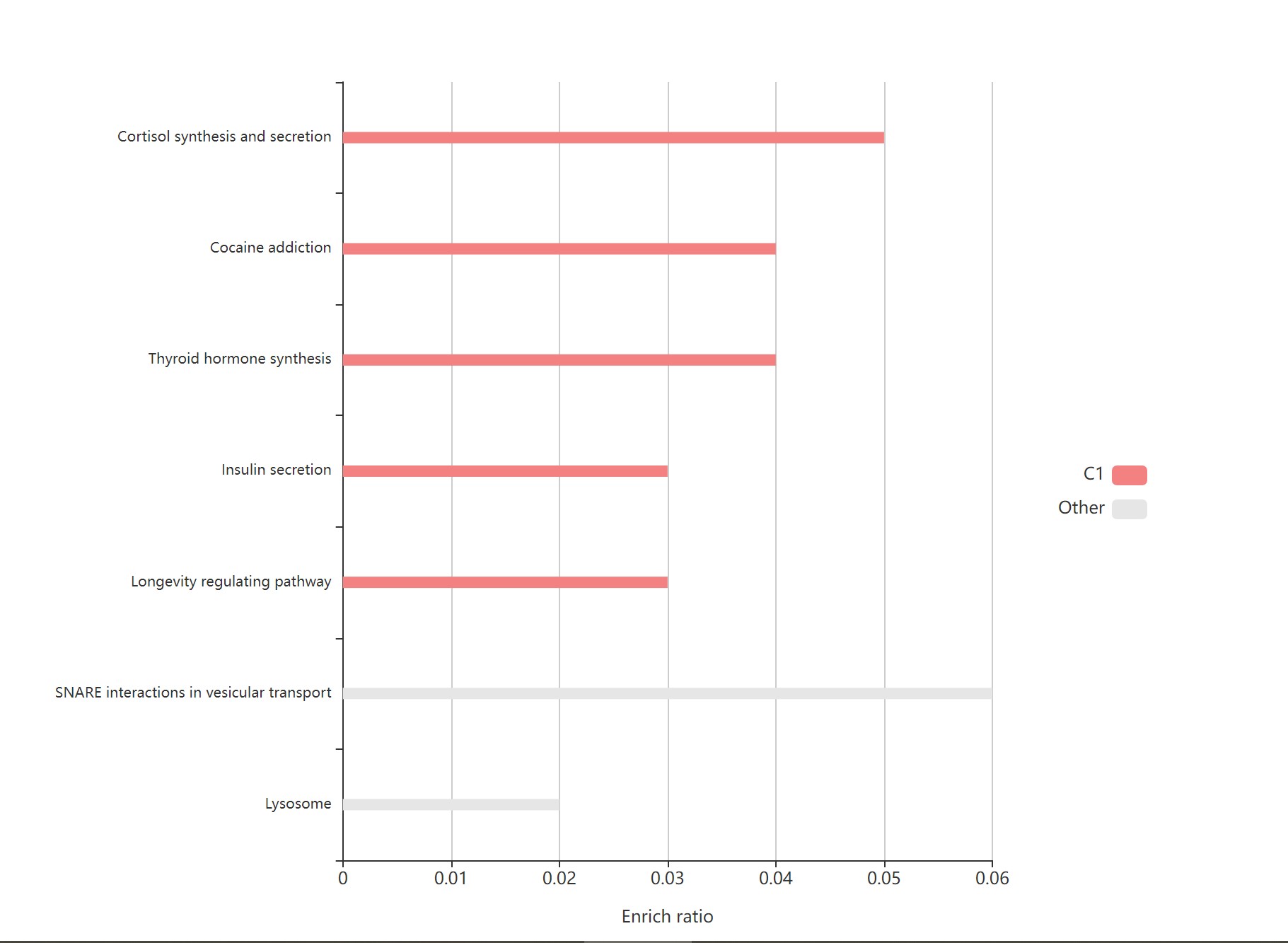


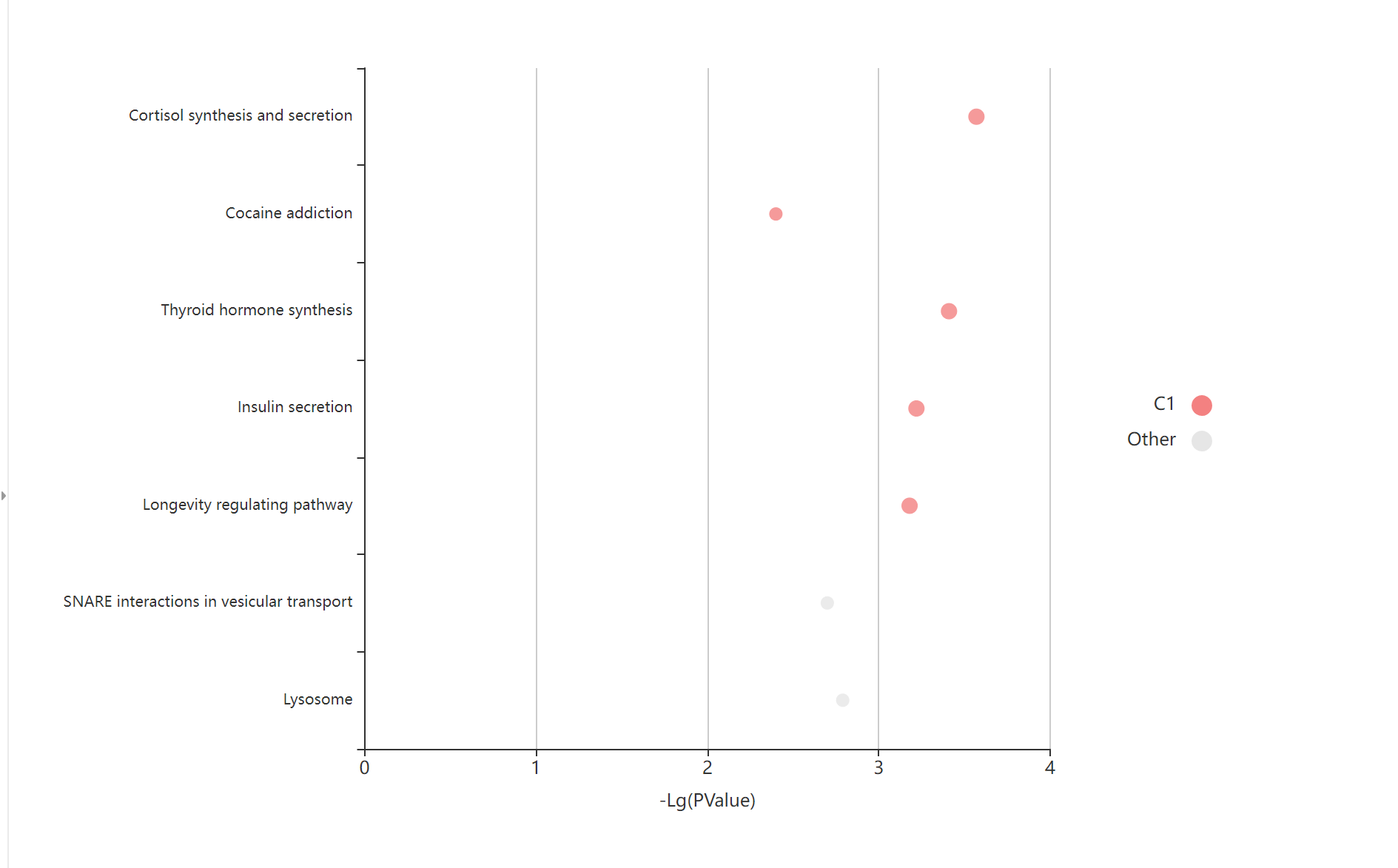


Fig S7. Enrichment analysis
